# Relationship between Snail Population Density and Infection Status of Snails and Fish with Zoonotic Trematodes in Vietnamese Carp Nurseries

**DOI:** 10.1371/journal.pntd.0001945

**Published:** 2012-12-20

**Authors:** Jesper Hedegaard Clausen, Henry Madsen, K. Darwin Murrell, Van Phan Thi, Hung Nguyen Manh, Khue Nguyen Viet, Anders Dalsgaard

**Affiliations:** 1 Department of Veterinary Disease Biology, Faculty of Health and Medical Sciences, University of Copenhagen, Copenhagen, Denmark; 2 DBL Centre for Health Research and Development, Department of Veterinary Disease Biology, Faculty of Health and Medical Sciences, University of Copenhagen, Copenhagen, Denmark; 3 Centre for Environment and Disease Monitoring in Aquaculture (CEDMA), Research Institute for Aquaculture No.1, Bac Ninh, Vietnam; 4 Institute of Ecology and Biological Resources (IEBR), Vietnam Academy of Science and Technology, Hanoi, Vietnam; Khon Kaen University, Thailand

## Abstract

**Background:**

Fish-borne zoonotic trematodes (FZT) are a food safety and health concern in Vietnam. Humans and other final hosts acquire these parasites from eating raw or under-cooked fish with FZT metacercariae. Fish raised in ponds are exposed to cercariae shed by snail hosts that are common in fish farm ponds. Previous risk assessment on FZT transmission in the Red River Delta of Vietnam identified carp nursery ponds as major sites of transmission. In this study, we analyzed the association between snail population density and heterophyid trematode infection in snails with the rate of FZT transmission to juvenile fish raised in carp nurseries.

**Methodology/Principal Findings:**

Snail population density and prevalence of trematode (Heterophyidae) infections were determined in 48 carp nurseries producing Rohu juveniles, (*Labeo rohita)* in the Red River Delta area. Fish samples were examined at 3, 6 and 9 weeks after the juvenile fish were introduced into the ponds. There was a significant positive correlation between prevalence of FZT metacercariae in juvenile fish and density of infected snails. Thus, the odds of infection in juvenile fish were 4.36 and 11.32 times higher for ponds with medium and high density of snails, respectively, compared to ponds where no infected snails were found. Further, the intensity of fish FZT infections increased with the density of infected snails. Interestingly, however, some ponds with no or few infected snails were collected also had high prevalence and intensity of FZT in juvenile fish. This may be due to immigration of cercariae into the pond from external water sources.

**Conclusions/Significance:**

The total number and density of potential host snails and density of host snails infected with heterophyid trematodes in the aquaculture pond is a useful predictor for infections in juvenile fish, although infection levels in juvenile fish can occur despite low density or absence infected snails. This suggests that intervention programs to control FZT infection of fish should include not only intra-pond snail control, but also include water sources of allochthonous cercariae, i.e. canals supplying water to ponds as well as snail habitats outside the pond such as rice fields and surrounding ponds.

## Author Summary

Fishborne Zoonotic Trematodes (FZT) are an important public health challenge in Vietnam and other Asian countries where consumption of raw or inadequately cooked fish is part of the diet. Nearly half of the seafood consumed globally originates from aquaculture in particular produced in Asian countries. Therefore, control programs to prevent FZT infections in fish produced from aquaculture are needed for limiting or preventing these neglected fishborne diseases. During an investigation over two years on FZT infections in aquaculture nurseries, we found a positive correlation between potential snail host population size and FZT infection rates in fish. Clearly, the role of the snail vectors and their interaction with fish is crucial for development of effective control programs. However, snail populations external to the fish pond are also a potential source of cercariae, must be included in the design of FZT prevention programs for fish farms.

## Introduction

Production of fish for human consumption from aquaculture, have expanded rapidly and it is estimated that 48.2% of fish for human consumption now originates from aquaculture, a number that is expected to rise further [1]. Risks of fishborne zoonotic trematodes (FZT) infections in humans [2], [3] has been linked to the increase in aquaculture production [4], [5] and WHO added FZT to their list of emerging infectious diseases, i.e. clonorchiasis and opistorchiasis where it is estimated that these two diseases result in 275,370 and 74,367 “disability-adjusted life years” (DALY's) globally, with Vietnam accounting for 26,366 DALY's [5]. In Vietnam, fish is a main source of protein [6], and FZT infections in humans [7], [8] and fish [9], [10] are frequently reported. Cyprinid fish are common in aquaculture and well-known carriers of FZT. Integrated household-based culture systems that combine fish culture, raising pigs and horticulture are common in Vietnam and have been shown to have high risk for FZT transmission [11].

The public health aspects of infections with the liver flukes are severe and include life threating liver cancer [2], [3]. Diseases caused by the intestinal flukes (hetereophyid trematodes) are less well known, but clinical manifestations of infection in humans have been reported. Infections with Haplorchis can be responsible for significant morbidity [12] and cases from Thailand show that human infection with H. taichui can cause mucosal ulceration, mucosal and submucosal haemorrhages, fusion and shortening of villi, chronic inflammation, and fibrosis of the submucosa [13].

During fish culture there are two main intervention points to prevent FZT transmission, namely fecal pollution of the pond environment (trematode eggs) and introduction of cercariae in the aquaculture environment. Intervention studies conducted in Vietnam showed that controlling these two factors reduced FZT infections in juvenile fish [14]. Thus, the control of snails play a crucial role in the intervention strategies as certain snail species serve as intermediate host for the FZT. The potential host snails for trematodes of the family Heterophyidae are snails from the families Thiaridae, Bithynidae, and Stenothyridae [15].

Interventions aimed at controlling FZT infections in fish should take a holistic approach focusing on all stages of the transmission cycle [11], [14]. Preventing intermediate host snails from establishing populations in ponds and preventing them becoming infected during the culture period would be one important intervention. Mud and vegetation removal should be carefully done by farmers to reduce snail population density prior to stocking, and water-runoff management should prevent fecal matter being washed into the pond, hence reducing the probability that existing snails become infected. Filtering of water going into the pond should be done, but common practice of filtering water is often done with a filter mesh size too large to prevent smaller snails and cercariae from entering the pond. Depending on the frequency of refilling ponds during a production cycle, control of snails in the water supply habitat, e.g. small canals, could be important to reduce snail or cercariae contamination of ponds [14]. Knowledge on the relationship between snail density and FZT infection levels in snails with FZT infection in fish could help assess whether intervention against snails in the pond would be sufficient to control infections in fish.

The study objective was to analyze the association between snail population density and heterophyid trematode infection in snails with the prevalence and intensity of FZT transmission to juvenile fish raised in carp nurseries.

## Materials and Methods

### Study area and design

**Figure 1:**
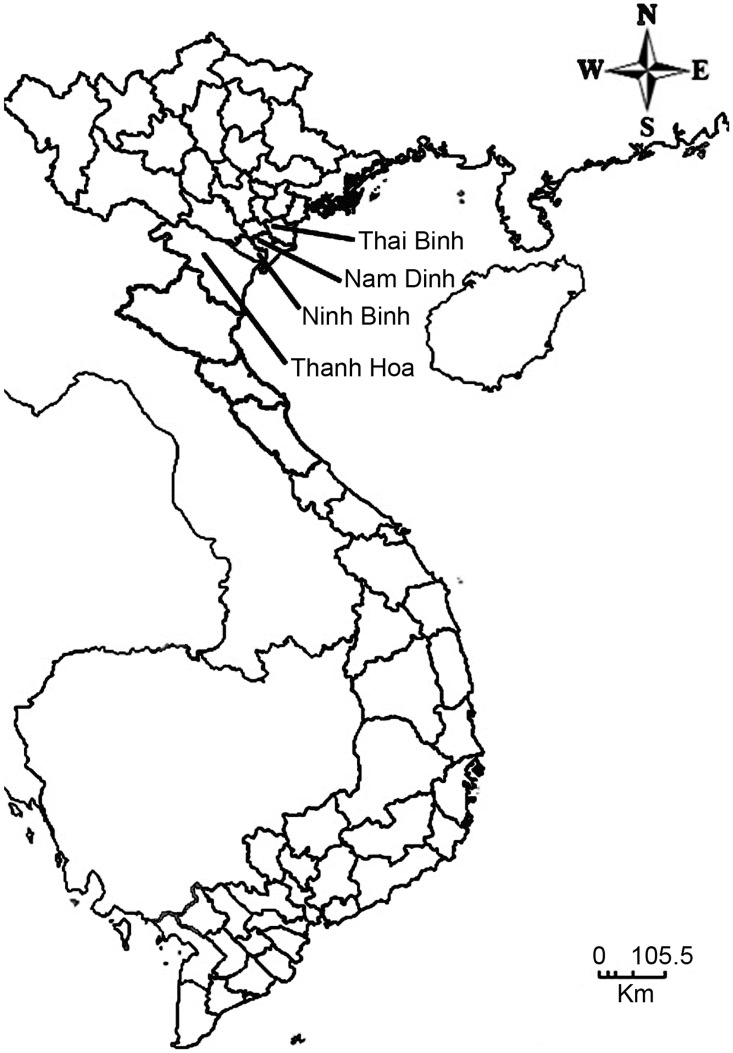
The four Red River Delta Provinces of Nam Dinh, Thai Binh, Ninh Binh and Thanh Hoa, Vietnam.

The study was conducted in Thai Binh, Nam Dinh, Ninh Binh and Thanh Hoa provinces all located in the Red River Delta of Northern Vietnam (Fig. 1). The provinces are known to have a high prevalence of fish-borne zoonotic trematodes (FZT) in humans, other animal reservoir hosts and fish and the provinces are all important aquaculture production areas [16], [17]. A total of 48 carp nurseries, 36 in 2009 and 12 in 2010 culturing Rohu (*Labeo rohita*) were selected for the study from a list of farms provided by the local Department of Agriculture and Rural Development. Rohu was selected because this species is known to be a potential host for FZT and is a major species raised by the farmers in the area during the time of year for this study. However, it is expected that associations between snails and FZT infections in fish will be similar for other fish species. The data on FZT infection levels in juvenile fish and snails was obtained as part of a larger intervention program and the study was designed as a parallel group design of nurseries with one intervention group and one non-intervention group [14]. The study unit was one nursery pond in each household which were all family driven nurseries. In this paper we analyze snail data and FZT infections in fish irrespective of whether interventions were done or not.

### Ethics statement

The study was done following the European Convention for the “Protection of Vertebrate Animals used for Experimental and Other Scientific Purposes”. Animals were handled with respect according to the study protocol which was approved by the Aquatic Animal Scientific Committee of Research Institute for Aquaculture No.1, under Ministry of Agriculture and Rural development, Vietnam.

### Snail sampling and examination for cercariae

For the collection of snails a special dredge was designed. The dredge was composed of an iron frame made of flat iron with a width of 0.25 m and a net bag made from nylon-mesh (mesh size about 1 mm) was attached. The edge of the nylon net was enforced by a double layer of cloth (20 mm wide) on each side of the net before made into a bag. A 3-m long handle was attached to the frame such that the angle between the frame and the handle could be adjusted depending on depth of the individual pond.

The dredge was used to scrape the pond bottom from 2 m from the pond bank to the edge of the pond at five different positions in each pond (i.e. the area sampled at each position was 2×0.25 m^2^ = 0.5 m^2^). The content of the dredge was emptied into a white tray and sorted for live snails that were transferred to a labeled container. Snail sampling was done 3, 6 and 9 weeks after stocking juvenile fish.

In the laboratory, snails were sorted to species and examined for infection by shedding or crushing. For the shedding method, smaller snails, including most of the species known to be intermediate hosts for FZT were placed individually in small plastic containers with 5 ml of water and left for 24 hours for shedding. Snails were checked for shedding late afternoon from 6.00–8.00 pm and again the following morning from 6.00–8.00 am. The advantage of this method is that cercariae obtained are fully developed allowing for optimal morphological identification. The crushing method involves crushing whole snails between two glass plates and checking hemolymph and tissue for cercariae or rediae. Species of the Viviparidae and Ampullaridae were not found infected with either parapleurolophocercous or pleurolophocercous cercariae (the types produced by Heterophyidae and Opistchorchidae;) in previous studies [15]. Dung et al. 2010 [15] further found these cercariae in all common species of Thiaridae in Nam Dinh Province and they were also found in Bithynidae (Bithynia fuchsiana). Therefore crushing and shedding and analyses are only done on cercariae data obtained from examination of Thiaridae and Bithynidae.

Cercariae were identified to morphotype according to keys by Ginetsinskaya [18] and Shell [19]. The morphotype for FZT are the pleurolophocercous cercariae, which could be further divided into two categories, i.e. pleurolophocercous and parapleurolophocercous cercariae depending on whether lateral finfolds are present on the tail in addition to the dorso-ventral finfold [19]. Pleurolophocercous cercariae are produced in the families' Heterophyidae, Cryptogonimidae and Opisthorchiidae, while parapleurolophocercous cercariae are found within the Heterophyidae. All “pleurolophocercous” cercariae retrieved within this study were of the parapleurolophocercous type; this distinction is important because the liverfluke *Clonorchis sinensis*, which have the pleurolophocercous cercariae, is found in the area as well. Thus we are certain that the cercariae identified in this study belong to the Heterophyidae. There may, however, be species among them that are not zoonotic, but these species would also use fish as second intermediate host. The Heterophyidae includes many of the important species of FZT's in Vietnam, i.e. *Haplochis pumilio*, *H. taichui*, *H. yokogawai*, *Centrocestus formosanus* and *Stellanchasmus falcatus* [13], [14], [20]–[22].

### Juvenile fish sampling and examination for metacercariae

To confirm that the fry stocked were free from FZT, 100 fry were sampled from the supplying hatchery and examined for metacercariae by the compression method [23]. At 3, 6 and 9 weeks after stocking, 100 juvenile fish from each pond were randomly collected using a feeding tray for first sampling and a seine net for the last two samplings. The fish were placed on ice and transported within 48 hours to the laboratory and maintained at 4°C for a maximum of 5 days until processed. Each individual fish was then ground with a small hand held homogenizer and transferred to a beaker for digestion in a pepsin solution consisting of 8 ml concentrated HCl, 6 g pepsin in 1,000 ml of water to release metacercariae [10]. After digestion, metacercariae were isolated by sediment washing and were identified and counted under microscope. For each juvenile fish, the number of metacercariae was counted and sorted into one or more type groups: liver flukes (Opisthorchiidae), intestinal flukes (Heterophyidae) and non-zoonotic or dead metacercariae. Metacercariae were identified using morphological criteria described previously [14].

### Statistical analysis

Data were double-entered in a ACCESS database (Microsoft, Redmond, WA, USA) and analyzed using Stata 12 (StataCorp LP, USA). In order to relate infections in fish (prevalence and intensity of infection) to density of intermediate host snails or of infected snail hosts, we averaged the snail density over the three sampling times. Density of infected intermediate host snails was divided into three categories (mean of the three samplings), i.e. Low (0 snails infected), Medium (0.14–1.07 snail infected m^−2^) and High (>1.07 infected snails m^−2^) and population density of intermediate host snails was categorized to these groups: Low (0 snails), Medium (0.14–13.3 snails m^−2^) and High (>13.3 snails m^−2^). Association between prevalence of infection in fish was analyzed with these snail density categories and reported as odds ratios after adjusting for year, stage of sampling (3, 6 and 9 weeks post-stocking) and clustering within ponds using logistic regression analysis [24]. Intensity of infection in fish was analyzed in a similar analysis using negative binomial regression [25], i.e. in generalized linear model with log-link function, and reported as count ratios. The ancillary parameter was estimated using full maximum likelihood estimation and over-dispersion was assessed using the dispersion statistics [25]. Model fit was evaluated using the Hosmer-Lemeshow goodness of fit statistic and residual analysis [24].

## Results

### Snails

**Figure 2:**
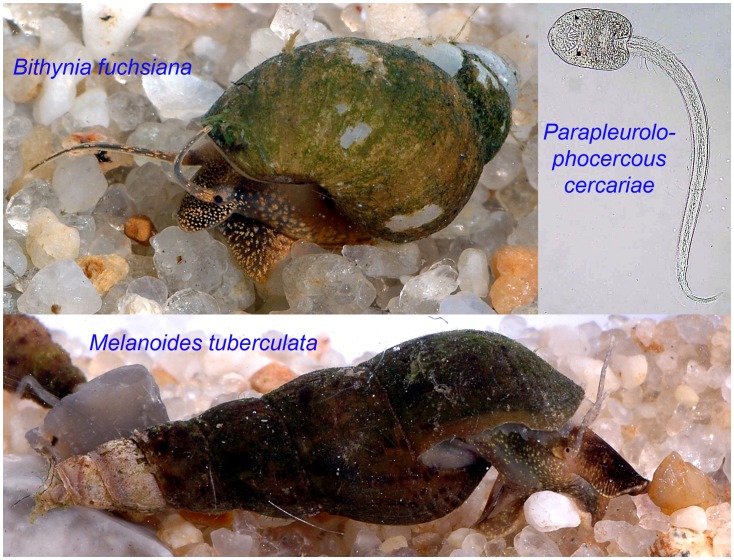
Two important snail intermediate hosts, *Bithynia fuchsiana* and *Melanoides tuberculata* and a Parapleurolophocercous cercaria (Photos by Henry Madsen).

A total of 26,979 snails were collected during the study (Table 1). Thiarid snails were the most abundant in the nursery ponds followed by viviparid snails, while bithynid and pulmonate snails were less common. Species of the Thiaridae and Bithynidae are known potential intermediate hosts for FZT (Fig. 2) [15]. Echinostome cercariae were also found in the Thiaridae and pulmonate (*Lymnaea* spp.) snails. We did find different morphotypes of the parapleurolophocercous cercariae (Fig. 2), but could not identify these to any particular species within the Heterophyidae.

**Table 1:** Total snails collected and prevalence of infected snails with parapleurolophocercous, echinostome and other cercarial types.

Snail family	Snails collected (n)	Parapleurolophocercous cercariae (%)	Echinostome cercariae (%)	Other cercariae (%)
Thiaridae^a^	14,343	2.1	0.1	3.5
Bithynidae^b^	1,104	0.2	0	3.1
Viviparidae^c^	10,580	n.d.^f^	n.d.	n.d.
Ampullariidae^d^	377	n.d.	n.d.	n.d.
Pulmonata^e^	575	0	0.9	0.9

a Including: *Thiara scabra*, *Tarebia granifera*, *Melanoides tuberculata* and *Sermyla riquetii*.

b Including: *Bithynia* spp. and *Parafossarulus striatulus*.

c Including: *Angulyagra polyzonata* and *Sinotaia aeruginosa*.

d Including: *Pila polita and Pomacea canaliculata*.

e Mainly *Lymnaea viridis* but also including *L. swinhoei* and *Gyraulus convexiusculus*.

f “n.d.” indicate that shedding and crushing was “not done”.

### Fish

**Figure 3:**
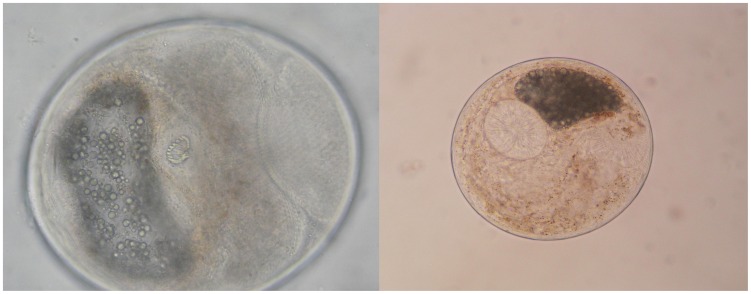
Metacercariae of *Haplorchis taichui* (left) and *Clonorchis sinensis* (right) (Photos by RIA1).

The total number of juvenile fish analyzed during the study was 16,850. From those, a total of 39,312 metacercariae were isolated and separated into four types (Table 2). For the subsequent data analysis only the data on Heterophyidae and Opisthorchiidae (Fig. 3) metacercariae were used. Other metacercariae found could either not be identified or were non-zoonotic.

**Table 2:** Number of metacercariae found in juvenile fish collected May–September, 2009 and May–September, 2010.

Metacercariae group	Total (n)	Prevalence (%)	Intensity (metacercariae/positive fish)
Hetereophyidae	36,959	20.0	11.05
Ophistorchiidae	11	0.1	2.20
Other^a^	1,146	3.0	2.26
Dead^a^	1,196	3.0	2.55

a The Other metacercariae group included those metacercariae that were identified as non-zoonotic (e.g. *Exorchis* spp.). Dead metacercariae were not identifiable because of the loss of morphological clarity. Neither of these two groups of metacercariae were included in the data analysis.

### Association between snail and fish data

**Figure 4:**
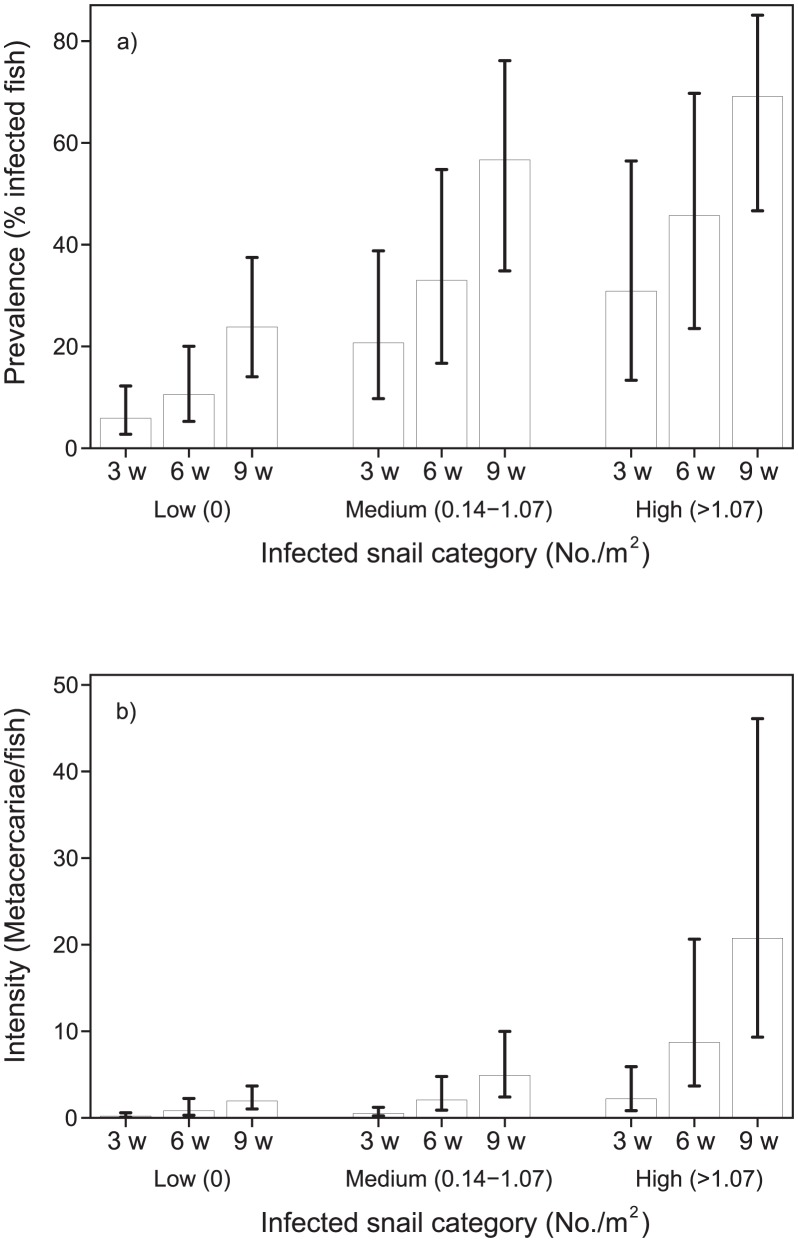
Relationship between FZT infected snails and prevalence (4a) and intensity (4b) of metacercariae in fish.

Figure 4 a) shows FZT prevalence (%) in fish and Figure 4 b) shows intensity (metacercariae/whole fish). Both prevalence and intensity is shown against three categories of snails as infected snails/m^2^, Low (0), Medium (0.14–1.07) and High (>1.07) at each of the three samplings at week 3, 6 and 9. The bars indicate 95% CI. Both the average prevalence and average intensity of infection in fish increased with density of infected snails and over time (Fig. 4) but the variation between ponds within each of the three categories for density of infected snails was very high (Table 3).

**Table 3:** Associations between potential snail hosts and infected snails with prevalence and intensity of FZT in fish^a^.

	Number of ponds	Prevalence in % (Range)	Intensity (Range)	Prevalence: Odds Ratio with 95% CI	Intensity: Count Ratio with 95% CI
**Density category of infected snails (snails m^−2^)**					
0 (Low)	34	23 (0–94)	0.19 (0–1.38)	1	1
0.14–1.07 (Medium)	8	54 (0–88)	0.55 (0–1.43)	4.36* (1.65–11.55)	2.37 (0.85–6.56)
>1.07 (High)	5	75 (30–97)	1.68 (0.28–5.09)	11.32* (4.46–28.76)	11.94* (3.89–36.66)
**Density category of snails (snails m^−2^)**					
0 (Low)	6	27 (0–86)	0.21 (0–0.96)	1	1
0.14–13.3 (Medium)	24	17 (0–84)	0.13 (0–1.22)	0.57 (0.10–3.08)	2.02 (0.45–8.96)
>13.3 (High)	17	60 (0–97)	0.83 (0–5.09)	4.01* (0.84–19.16)	18.41* (4.25–79.65)

* Indicates significance (P<0.001).

a For data on prevalence of FZT in fish results are shown as odds ratios and for intensity of FZT in fish as count ratios. “No infected snails” is set at value 1 and all other ratios are in relation to the “No infected snails”.

**Figure 5:**
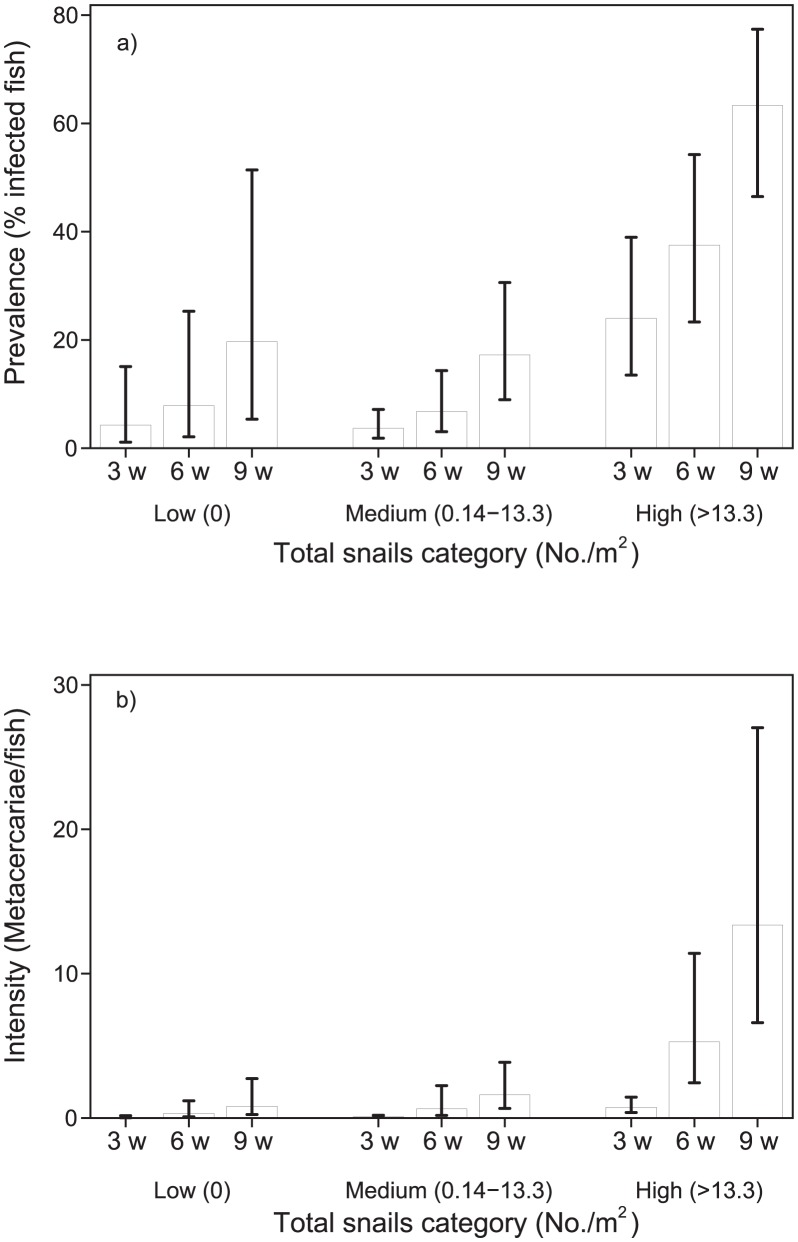
Prevalence (5a) and intensity (5b) of metacercariae in fish and its relation to potential snail host density.

Similarly, both prevalence and intensity of infection on average increased with density of snails and over time (Fig. 5). Prevalence (%) is shown Figure 5 a) and intensity (metacercaria/whole fish) in Figure 5 b). Both prevalence and intensity is shown against three categories of total snails/m^2^, Low (0), Medium (0.14–13.3) and High (>13.3) at each of the three samplings at week 3, 6 and 9. The bars indicate 95% CI. Variation of infection within each category of snail density was considerable (Table 3). Thus, the FZT prevalence in fish from two nurseries which had no infected snails was 86% and 94% (Table 3).

The sampling of snails outside two nursery ponds showed that all five samples were found to contain snails infected with heterophyids. From a total of 48 nursery ponds, snails from 21 were not infected with heterophyids although the prevalence of heterophyid FZT in fish was high.

### Analyses of association between snail and fish infections

In Table 3, the odds ratios and count ratios for prevalence and intensity of FZT in fish and their interactions with density of snails and infected snails are presented. The analyses were done after adjusting for year, sampling time (3, 6 and 9 weeks) and clustering within ponds.

There was a significant (P<0.001) association between prevalence of FZT in juvenile fish and density of infected snails in the ponds with the odds for infection in juvenile fish being 4.36 and 11.32 higher in the Medium and High infected snail categories, respectively when compared with the Low category group of infected snails. The correlation between increase in FZT intensity in fish and density of potential host snails was significant (P<0.001) with an odds ratio of 4.01 for ponds with the highest density of snails (Table 3).

The association between the increase in the density of infected snails in the ponds and the increase in intensity of FZT in fish was significant (P<0.001) with a count ratio of 11.94 for ponds with high density of infected snails. Similarly, an increase in density of snails was associated with an increase in FZT intensity in fish, i.e. the count ratio was 18.41 if 13.3 snails/m^2^ or more were found inside the pond (Table 3).

## Discussion

There was a significant correlation between FZT infected juvenile fish from aquaculture ponds and the population density of potential host snails and density of heterophyid infected snails residing in the ponds, underlining that any integrated intervention program must include measures to control snail vectors. The data obtained in this study adds important information on the association between snails and transmission of intestinal FZT, a relationship that is likely also to be important for the zoonotic liver flukes [26].

The FZT transmission pattern, however, is complicated and not always predictable as highlighted by our finding of high FZT prevalence in fish from some ponds despite the apparent absence of infected snails, e.g. in 21of 48 ponds with high FZT prevalence, no heterophyid infected snails were found. A possible explanation for this finding is that these ponds were contaminated with cercariae from sources located outside the ponds, e.g. cercariae present either in pond replenishment water obtained from canals harboring infected snails or introduced during flooding events [14]. Aquaculture ponds are often located adjacent to rice paddies, and irrigation canals. Heterophyid positive snails were often found in the snail samples collected outside the pond environment. The frequency of water exchange in the ponds with water from external sources, e.g. canals, may therefore also have an impact on snail densities in the pond through introduction of potential infected snails. This together with an abundance of potential host snails in these water bodies makes it difficult to establish effective biosecurity measures in nearby farm ponds. An alternative explanation for the high prevalence and intensity of FZT infected fish, is that infected snails were missed in the collected samples due to inadequate sampling procedures. We cannot rule out that infected snails were present in some ponds, but then this would have been in very low densities. This raises the question on whether a particular threshold of infected snail density exists necessary to maintain a high FZT transmission level in cultured fish. One snail may produce an average of 689 and up to more than 3,000 cercariae of *Haplorchis pumilio* per day [27], and because fish move around in the pond, the risk of exposure to cercariae can be high, compensating for a low prevalence of infected snails. A similarphenomenon of low host snail prevalence and high infection rates has been found for bilharzia in ecological studies [28], [29].

The heterophyid infections in snails may represent several species which may not all be zoonotic. Therefore, future studies with snails should include molecular tools for identification of trematode infections to species level [26]. It would strengthen results if molecular technique was used to help speciation of the cercariae. However, we used only morphology to identify cercariae and metacercariae; metacercariae could be identified to species while cercariae could only be identified to morphotype. To strengthen future studies even more, animal experimental life cycle studies can help in identification of these larval parasites. Since the scooping method is arather insensitive method to verify the presence of FZT infected snails, an alternative method could be to analyse water samples for cercarial DNA. However, this would not distinguish between autochtonous and allochtonous cercariae.

Our findings have direct implications for snail control. If a low-level of infection in snails can result in high level of infected fish, interventions against the intermediate host snails should be very effective even in the absence of contamination of the pond with allochtonous cercariae. To reduce contamination of ponds with cercariae, snail control should also be implemented in the water bodies that supply ponds with water or in areas adjacent to ponds where crops are grow that provides good snail habitats, e.g., rice fields. Treatment of water supplied to ponds may be done with filters such as sand filters if the water volume is not too large; an option that might be suitable for large commercial nurseries that want to market trematode-free fingerlings. It should be noted that implementation of sand filters would probably be a significant expense to a small-scale nursery operation, both in terms of instalment and maintenance cost. Further research are needed on the cost effectiveness of the different interventions, however this was beyond the scope of the current study. It is important to incorporate any changes as much as possible into exsisting good management programs as much as possible to limit new costs for farmers. Thus, control of water quality and prevention of surface runoff into ponds are critical for fish farms if prevention of FZT is to be achieved.
